# Global Trends in Vaccine Research on *Ehrlichia* and *Anaplasma*: A Bibliometric Review

**DOI:** 10.3390/microorganisms14091967

**Published:** 2026-09-06

**Authors:** Swetha Madesh, Chetan M Badgujar, Sreekumari Rajeev

**Affiliations:** 1 Department of Biomedical and Diagnostic Sciences, College of Veterinary Medicine, University of Tennessee, Knoxville, TN 37996, USA; smadesh@vols.utk.edu; 2 Department of Biosystems Engineering and Soil Sciences, University of Tennessee, Knoxville, TN 37996, USA; chetanmb@utk.edu

**Keywords:** *Ehrlichia*, *Anaplasma*, bibliometric analysis, tick-borne pathogens, antigen discovery, bovine anaplasmosis, heartwater

## Abstract

*Ehrlichia* and *Anaplasma* are important tick-borne obligate intracellular bacteria that cause clinically and economically significant infections in humans and animals. Despite their importance, vaccine development for these pathogens remains limited, and the overall progression of vaccine-related research in this field has not been quantitatively characterized. In this study, we conducted a comprehensive bibliometric analysis to characterize the trajectory and the research landscape of vaccine-related research for these pathogens. The literature was retrieved from the Web of Science Core Collection using the search terms (*Ehrlichia* OR ehrlichiosis OR *Anaplasma* OR anaplasmosis) AND (vaccine OR vaccines). A total of 483 publications published between 1965 and 2025 were included in the analysis. Bibliometric analyses were conducted using Biblioshiny (Version 4.3.3) and VOSviewer (Version 1.6.20) to assess publication trends, citation patterns, leading countries, institutions, and major research themes. The literature showed gradual growth over time, with more consistent publication output from the late 1990s onward. Scientific output was concentrated in a relatively small number of countries and institutions, led by the United States, South Africa, Brazil, Spain, and France. Keyword frequency and co-occurrence analyses showed that the field has been shaped mainly by bovine anaplasmosis, heartwater, and tick-associated vaccine research, with *Anaplasma marginale* and *Ehrlichia ruminantium* emerging as dominant pathogen-focused themes. Major surface proteins, msp1a, subolesin, and Bm86, appeared frequently, indicating continued interest in both pathogen-derived and tick-associated vaccine targets. In contrast, human and canine pathogens such as *Ehrlichia chaffeensis*, *Anaplasma phagocytophilum*, and *Ehrlichia canis* were less represented. Our analysis maps the research landscape of *Ehrlichia* and *Anaplasma* vaccine development, quantifying how research activity has been distributed across pathogens, geographic regions, institutions, collaborations, and vaccine-related themes. It also identifies areas that remain comparatively less represented in the published literature and provides a quantitative baseline for future comparisons with independent measures of disease burden and research funding data.

## 1. Introduction

Tick-borne diseases (TBDs) represent a significant and growing threat to both human and animal health worldwide. Ticks are among the most important arthropod vectors of infectious agents, transmitting a diverse array of bacterial, viral, and protozoan pathogens that cause many diseases of medical and veterinary importance [1,2]. The incidence and geographic distribution of TBDs have increased over recent decades due to factors such as climate and land use change, expansion of wildlife reservoirs, increased animal and human movement, and geographic expansion of tick vectors [3]. These diseases are associated with substantial morbidity, mortality, and economic losses resulting from healthcare costs, reduced livestock productivity, and the challenges of implementing effective tick control strategies [4]. Consequently, TBDs have emerged as an important One Health concern, highlighting the interconnectedness of human, animal, and environmental health.

Among the diverse bacterial pathogens transmitted by ticks, members of the family *Anaplasmataceae*, particularly the genera *Ehrlichia* and *Anaplasma*, are of major clinical and veterinary significance. These Gram-negative, obligately intracellular bacteria infect a wide range of vertebrate hosts and are transmitted primarily through ixodid ticks [5]. Different species exhibit distinct tropisms for specific host cell types, including monocytes and macrophages, neutrophils, platelets, endothelial cells, and erythrocytes, where they survive and replicate within membrane-bound vacuoles [6,7,8,9,10]. Infection can result in a spectrum of clinical outcomes ranging from subclinical persistence to severe and potentially fatal disease [6,7,8,9]. Clinically important species include *Ehrlichia chaffeensis*, *E. ewingii*, *E. canis*, and *E. ruminantium*, as well as *Anaplasma phagocytophilum*, *A. marginale*, *A. platys*, and *A. ovis* [5,6,7,8,9]. These pathogens are responsible for numerous emerging and endemic tick-borne diseases affecting humans, companion animals, and livestock worldwide. Their associated diseases, cellular tropisms, and host ranges are summarized in Table S1. Understanding the biology, epidemiology, and pathogenesis of these organisms is essential for improving disease surveillance, diagnosis, prevention, and control.

The medical and economic importance of these infections extends across human, companion-animal, and livestock health. In humans, *Ehrlichia chaffeensis* causes human monocytic ehrlichiosis (HME), while *Anaplasma phagocytophilum* causes human granulocytic anaplasmosis (HGA). Clinical manifestations range from mild febrile illness to severe disease characterized by thrombocytopenia, leukopenia, hepatitis, respiratory distress, neurological complications, and occasionally death, particularly in elderly and immunocompromised individuals [6]. In dogs, *Ehrlichia canis* is the causative agent of canine monocytic ehrlichiosis, an important tick-borne disease of companion animals worldwide. Infection may result in acute fever, lymphadenopathy, thrombocytopenia, hemorrhagic tendencies, anemia, chronic wasting, pancytopenia, and death [7]. *Anaplasma platys* causes canine cyclic thrombocytopenia and frequently occurs as a co-infection with *E. canis*, potentially complicating diagnosis and clinical management [10,11]. In livestock, *Anaplasma marginale* causes bovine anaplasmosis, a severe hemolytic disease characterized by anemia, jaundice, abortion, loss of condition, and death in susceptible cattle [9,12]. *Ehrlichia ruminantium*, the causative agent of heartwater, affects cattle, sheep, and goats and is associated with high mortality rates in susceptible populations. These infections remain major obstacles to livestock health in many tropical and subtropical regions [8,9,12,13].

The economic impact of *Ehrlichia* and *Anaplasma* infections is substantial because of their effects on livestock production, veterinary care costs, and disease control measures [4,12,13,14]. *Anaplasma marginale* is regarded as one of the most economically important tick-borne pathogens of cattle worldwide. Infected herds experience losses attributable to mortality, reduced weight gain, decreased milk production, reproductive failure, treatment expenses, and trade restrictions [12,15]. Annual losses to the U.S. cattle industry have historically been estimated at more than USD 300 million [15]. Heartwater caused by *E. ruminantium* similarly exerts major economic impacts in endemic areas through high mortality rates, reduced productivity, and limitations on livestock movement and genetic improvement programs. It remains one of the most important tick-borne diseases of ruminants in sub-Saharan Africa and some Caribbean regions [8]. Heartwater also imposes a substantial economic burden on endemic livestock systems in South Africa, with estimated annual losses of approximately 1.27 billion South African rand across cattle, sheep, and goats [16]. In companion animals, ehrlichiosis and anaplasmosis contribute to substantial veterinary expenditures associated with diagnostics, long-term antimicrobial therapy, hospitalization, blood transfusions, and management of chronic disease, while persistent and chronic infections can further increase treatment requirements and negatively affect animal welfare [7,10]. Beyond direct disease-associated losses, high economic costs arise from tick-control programs, including acaricide use, surveillance, and preventive management strategies.

Diagnosis of *Ehrlichia* and *Anaplasma* infections involves clinical and hematological evaluation together with laboratory methods, including microscopic examination of blood smears, serological testing, and molecular methods such as polymerase chain reaction (PCR). Microscopic detection of intracellular morulae can be useful during acute infection when circulating organisms are more abundant, whereas serological testing often requires paired acute and convalescent phase samples to support diagnosis, and PCR can provide earlier detection of pathogen DNA during acute infection [6,17]. In humans, an indirect immunofluorescence assay (IFA) is commonly used for serological diagnosis, whereas in dogs, an IFA and rapid in-clinic antibody tests are widely used for detecting antibodies against *Ehrlichia* and *Anaplasma* species [10,17,18]. A PCR is useful for detecting DNA and differentiating infecting species [6,19]. Bovine anaplasmosis is commonly diagnosed using a blood smear examination, serological and molecular methods, with a competitive enzyme-linked immunosorbent assay (cELISA), widely used to detect antibodies against *A. marginale,* and a PCR is used for direct detection of pathogen DNA, including in persistently infected carrier cattle [15]. Heartwater is primarily diagnosed by PCR-based detection of *E. ruminantium*, supported by clinical and pathological findings, while microscopic demonstration of organisms in vascular endothelial cells can provide additional confirmation [8]. Despite advances in diagnostic testing, timely and accurate diagnosis can remain challenging because the sensitivity and interpretation of available methods are influenced by the stage of infection, pathogen load, antibody response, and clinical presentation [6,15,19].

Current control of *Ehrlichia* and *Anaplasma* infections relies largely on tick control and antimicrobial therapy. Acaricides are widely used to control tick vectors, but their continued use is increasingly constrained by cost, environmental concerns, reduced efficacy, and the emergence of acaricide resistance [20]. Treatment of *Ehrlichia* and *Anaplasma* infections is based primarily on tetracycline-class antimicrobials, with doxycycline commonly used in humans and dogs and oxytetracycline used for bovine anaplasmosis and heartwater [6,7,8,15]. Although these antimicrobials are generally effective for controlling clinical disease, particularly when initiated early, treatment may not always eliminate persistent infection [7,9,15]. Together, the substantial health and economic burden of these infections, challenges in diagnosis, and limitations of existing control and treatment strategies highlight the continued need for effective preventive approaches, including vaccination [21,22,23].

Vaccination offers a promising and potentially sustainable approach for control, but the development of effective vaccines against *Ehrlichia* and *Anaplasma* remains difficult because of the complex biology of these pathogens. Major challenges include their obligate intracellular nature, antigenic and strain diversity, variation in host immune responses, incomplete understanding of protective immune mechanisms, and difficulties in identifying broadly protective antigens [21,22,24].

Vaccine development against *Ehrlichia* and *Anaplasma* has a long history, particularly for livestock pathogens. Vaccination against bovine anaplasmosis dates to the early twentieth century, when the less virulent *Anaplasma centrale* was introduced as a live vaccine to reduce the severity of disease caused by *A. marginale*; this approach continues to be used in several endemic regions [9,23,25]. Heartwater vaccination also has a long history, with the traditional live infection-and-treatment approach involving inoculation of animals with *E. ruminantium*-infected blood followed by tetracycline treatment [8,26]. Although these live vaccine approaches have contributed to disease control, their use is limited by variable cross-protection, safety concerns associated with live or blood-derived vaccines, and practical requirements for production, storage, and administration [26]. These limitations have driven the development of safer and more broadly protective alternative vaccine strategies, including attenuated organisms, inactivated vaccines, recombinant and subunit antigens, DNA vaccines, and genetically modified live vaccine candidates [21,22,23]. In contrast, no commercial vaccine is currently available for canine monocytic ehrlichiosis caused by *E. canis*, human ehrlichiosis caused by *E. chaffeensis* or *E. ewingii*, granulocytic anaplasmosis caused by *A. phagocytophilum*, canine cyclic thrombocytopenia caused by *A. platys* [21,22,27,28]. Experimental vaccine development has progressed to different extents among these pathogens, with recombinant antigen, attenuated, and genetically modified live vaccine approaches investigated for *E. canis* and *E. chaffeensis*, while recombinant and genetically modified live vaccine approaches have been evaluated for *A. phagocytophilum* [21,27,29,30]. Research on *A. platys* and *A. ovis* remains at earlier stages, focused primarily on the identification and evaluation of potential vaccine targets [31,32].

Despite the challenges, vaccine-related research on *Ehrlichia* and *Anaplasma* has continued for several decades, generating a substantial and diverse body of literature spanning human, companion-animal, and livestock pathogens; multiple vaccine platforms and antigen targets; and research conducted across different geographic regions and institutions. Individual studies and reviews have provided important insights into specific pathogens, vaccine candidates, and mechanisms of protective immunity [21,22,23]. However, how this research area has evolved over time, which pathogens and vaccine approaches have received the greatest research attention, and how research activity is distributed globally have not been quantitatively characterized. The dispersed nature of this literature makes it difficult to obtain an integrated view of the field and to identify broader patterns across pathogens, vaccine approaches, and research activity.

Unlike systematic reviews, which synthesize evidence to address a focused research question, bibliometric analysis evaluates the scientific literature and provides a quantitative framework for mapping scientific output and identifying research trends. It is particularly useful for examining the extensive scientific literature over extended periods and for identifying broader patterns in the development, distribution, and organization of research activity within a field [33,34]. This makes bibliometric analysis well suited to characterizing the trajectory and structure of the published literature.

In this study, bibliometric analysis was applied to quantitatively characterize approximately six decades of *Ehrlichia* and *Anaplasma* vaccine research and examine how research activity has been distributed across time, geographic regions, institutions, collaborations, and major vaccine-related themes. This analysis provides a quantitative bibliometric baseline for understanding how the research landscape has evolved, identifying areas of concentrated and comparatively limited research activity, and supporting future comparisons of publication patterns with epidemiological, economic, disease-burden, and research funding data.

## 2. Materials and Methods

### 2.1. Data Collection and Search Strategy

**Figure 1:**
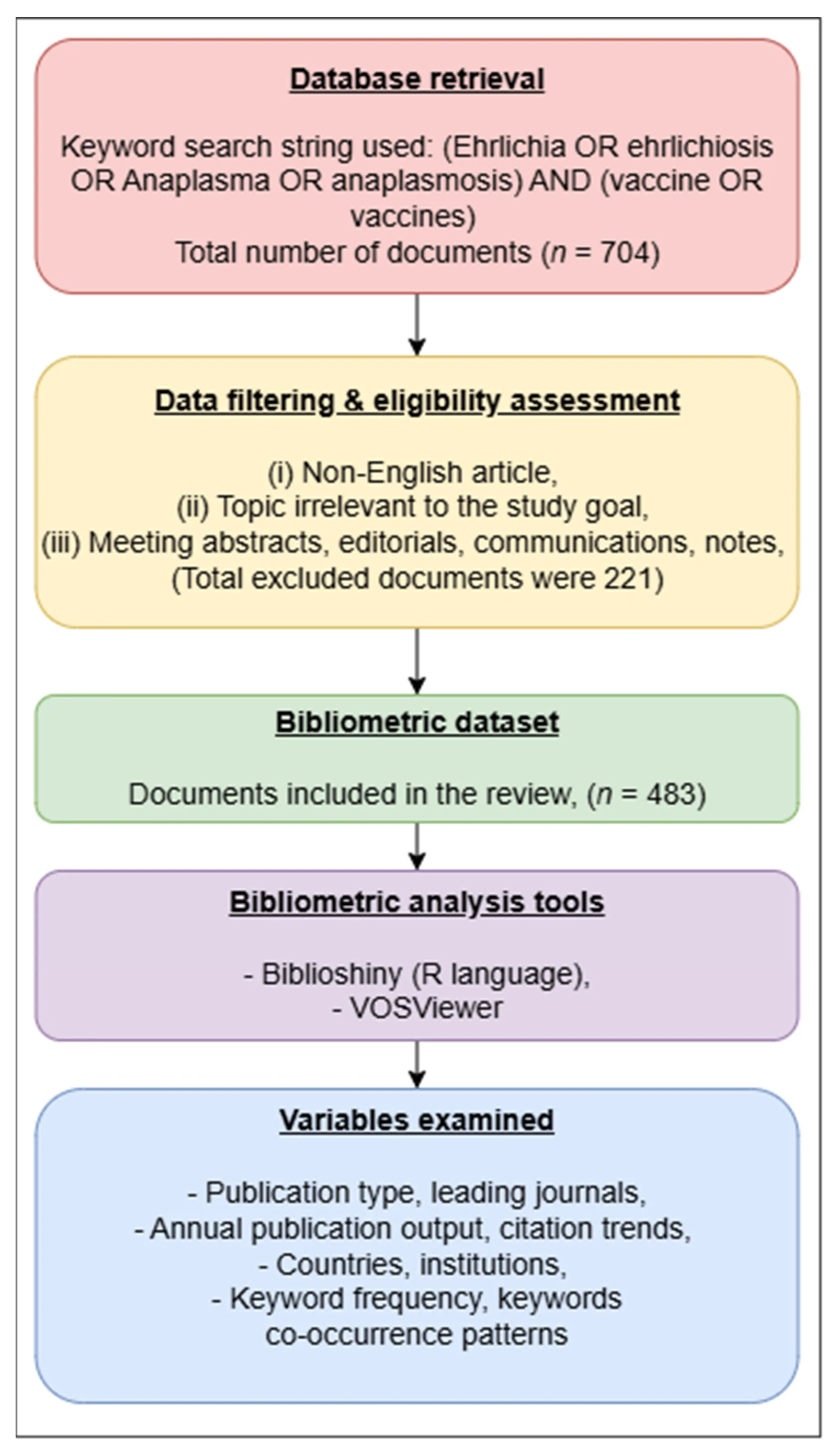
Overview of the workflow used for bibliometric analysis.

The bibliometric dataset was retrieved from the Web of Science database, a widely used source for literature retrieval and bibliographic analysis [35]. A keyword-based search strategy was applied using terms related to pathogens, associated diseases, and vaccines to identify the relevant publications. The search string used was (*Ehrlichia* OR ehrlichiosis OR *Anaplasma* OR anaplasmosis) AND (vaccine OR vaccines). This strategy allowed the inclusion of studies that referred either to the pathogens or to the diseases they cause, while limiting the dataset to the vaccine-focused literature. The retrieved records were exported to a CSV file, which was then screened and subjected to bibliometric processing. The overall workflow is presented in Figure 1.

### 2.2. Data Screening

The retrieved records were manually screened based on the publication title, abstract, and author keywords to determine their relevance to *Ehrlichia* or *Anaplasma* vaccine research. Publications were considered relevant when the primary focus involved vaccine development or evaluation, including identification or evaluation of vaccine antigens, vaccine-induced immune responses, and anti-tick vaccination approaches. Records were excluded when vaccine-related terminology was mentioned only in the discussion or keywords, but the primary focus of the study related to epidemiology, diagnosis, pathogenesis, treatment, or unrelated pathogens. Publication types such as meeting abstracts, editorials, notes, letters, and brief conference proceedings were excluded. These publication formats are often brief, preliminary, and may not provide the complete scientific and bibliographic information available in peer-reviewed full-length publications. In contrast, proceedings articles were retained when the conference contribution was published as a research article and met the relevance criteria for *Ehrlichia* or *Anaplasma* vaccine research.

### 2.3. Bibliometric Analysis

The final screened dataset was analyzed using Biblioshiny (Version 4.3.3) in R (Version 4.5.1) and VOSviewer (Version 1.6.20) [33,36]. These tools are widely used in bibliometric research to organize bibliographic records, generate descriptive bibliometric indicators, and visualize relationships among publications and keywords [34]. The bibliometric indicators used in this study included an assessment of publication output, citation trends, leading journals, institutions, and countries. Keyword frequency and co-occurrence analyses were performed using author keywords to identify recurring terms and major research clusters within the field. After the initial keyword analysis, plural forms, spelling variations, synonyms, and abbreviations were consolidated using a predefined synonym list, and the analyses were repeated to provide a more consistent representation of the keyword patterns.

## 3. Results

### 3.1. Data Overview

The initial search yielded 704 publications from the Web of Science database. After applying the exclusion criteria, 483 publications were retained for bibliometric analysis. The final bibliometric dataset comprised 483 documents published between 1965 and 2025 from 161 sources. Among the 161 unique publication sources, 144 (89.4%) were journals, 10 (6.2%) were proceedings-based publication sources, and seven (4.3%) were books. The field showed an annual growth rate of 4.08%, indicating a steady growth in publication output over time. The average document age was 15.7 ± 11.1 years, suggesting that the dataset was not limited to recent publications. A total of 1809 authors contributed to the included publications, with an average of 6.46 ± 3.80 co-authors per document. In addition, 173 (35.82%) publications involved international co-authorship, indicating active collaboration across countries in *Ehrlichia* and *Anaplasma* vaccine research. The dataset also included 1290 database-generated keywords and 849 author keywords. A summary of the data overview is shown in Table 1.

**Figure 2:**
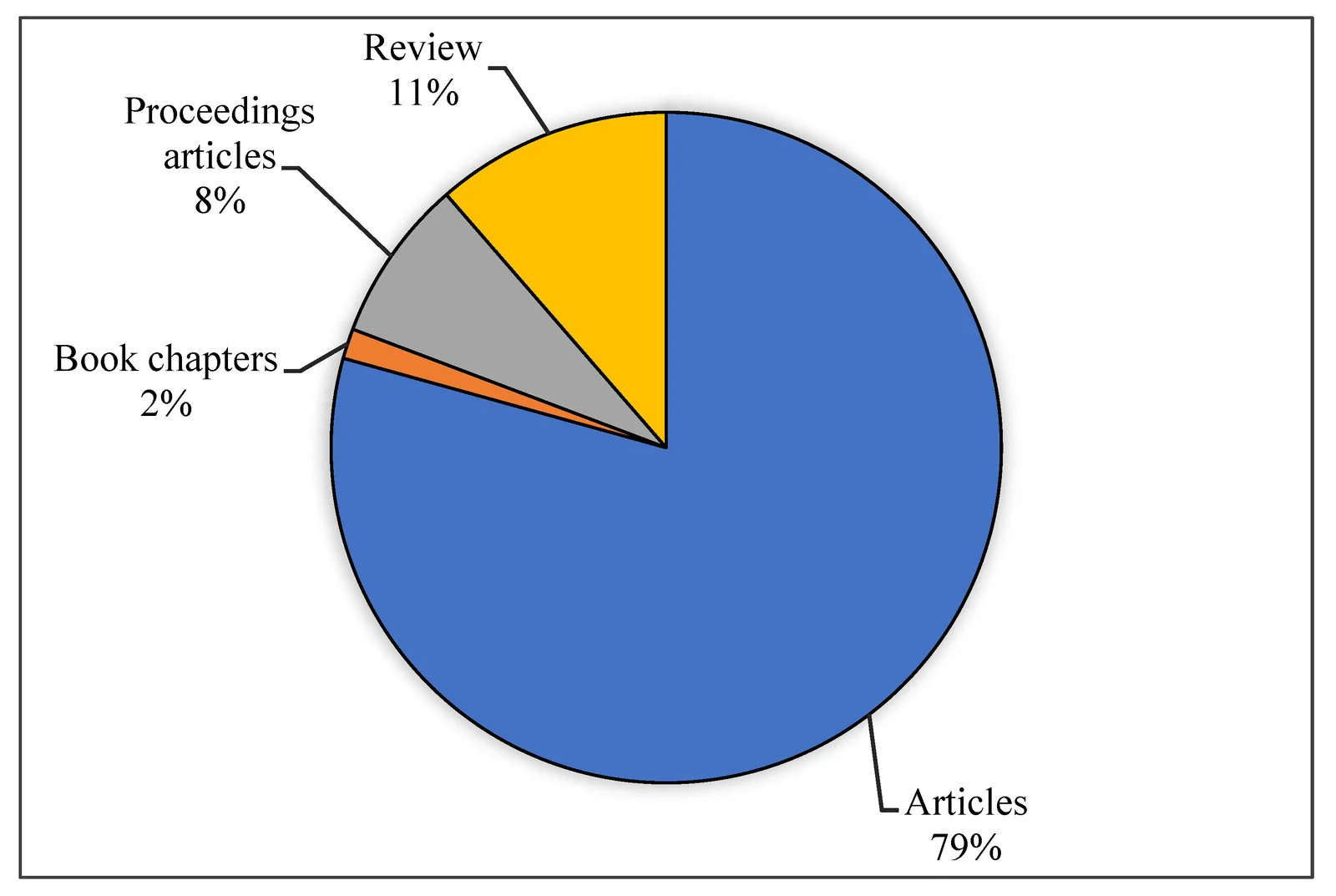
Distribution of document types of publications included in the bibliometric dataset.

The distribution of document types showed that original research articles represented the largest proportion of the literature (*n* = 383, 79%), followed by review articles (*n* = 55, 11%), proceedings articles (*n* = 38, 8%), and book chapters (*n* = 7, 2%) (Figure 2). The pattern indicates that research in this field has been primarily driven by original studies. Review articles accounted for a smaller proportion of the published literature, suggesting that fewer studies have focused on synthesizing the available literature. Proceedings articles also indicate that some findings have been reported through scientific meetings.

### 3.2. Annual Publication and Citation Trends

**Figure 3:**
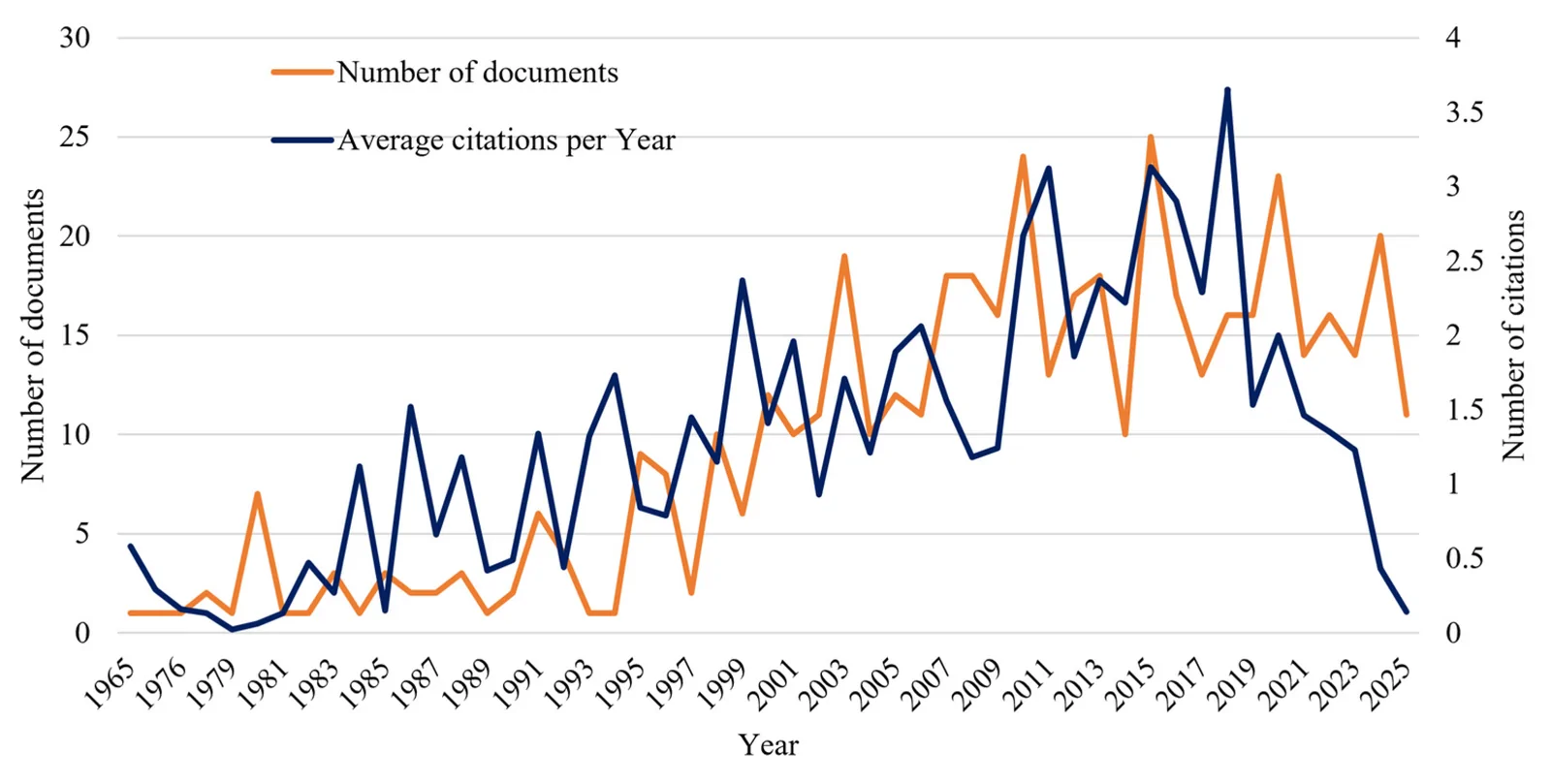
Annual publication output and average citations per year in *Ehrlichia* and *Anaplasma* vaccine research.

The annual publication pattern showed that research on *Ehrlichia* and *Anaplasma* vaccine studies has a long publication trajectory but remained limited for many years, with only a few documents published annually in the earlier decades (Figure 3). Publication output became more consistent from the late 1990s onward and increased further after 2000, although year-to-year fluctuations were still observed. Overall, the pattern indicates a gradual increase in research output over time. This trend is consistent with the broader literature, which suggests that vaccine development for *Ehrlichia* and *Anaplasma* has progressed gradually because of biological and technical challenges, including antigenic variation and difficulties in identifying protective vaccine targets [22,23,24,27]. The reduced number of publications in 2025 likely reflects incomplete database indexing at the time of analysis.

Citation patterns also varied across years, with several peaks observed during the study period, particularly in later periods (Figure 3), suggesting differences in citation activity across publication years. Average citations per year were calculated by dividing the mean total citations per article by the number of years available for citation accumulation through 2025. This calculation accounts for differences in the time available for publications to accumulate citations, but it does not account for changes in the number of publications or the number of references cited in scientific articles over time. Lower citation values in most recent years may still be expected because these publications have had relatively limited time to accumulate citations.

### 3.3. Geographic Distribution and Collaboration

**Figure 4:**
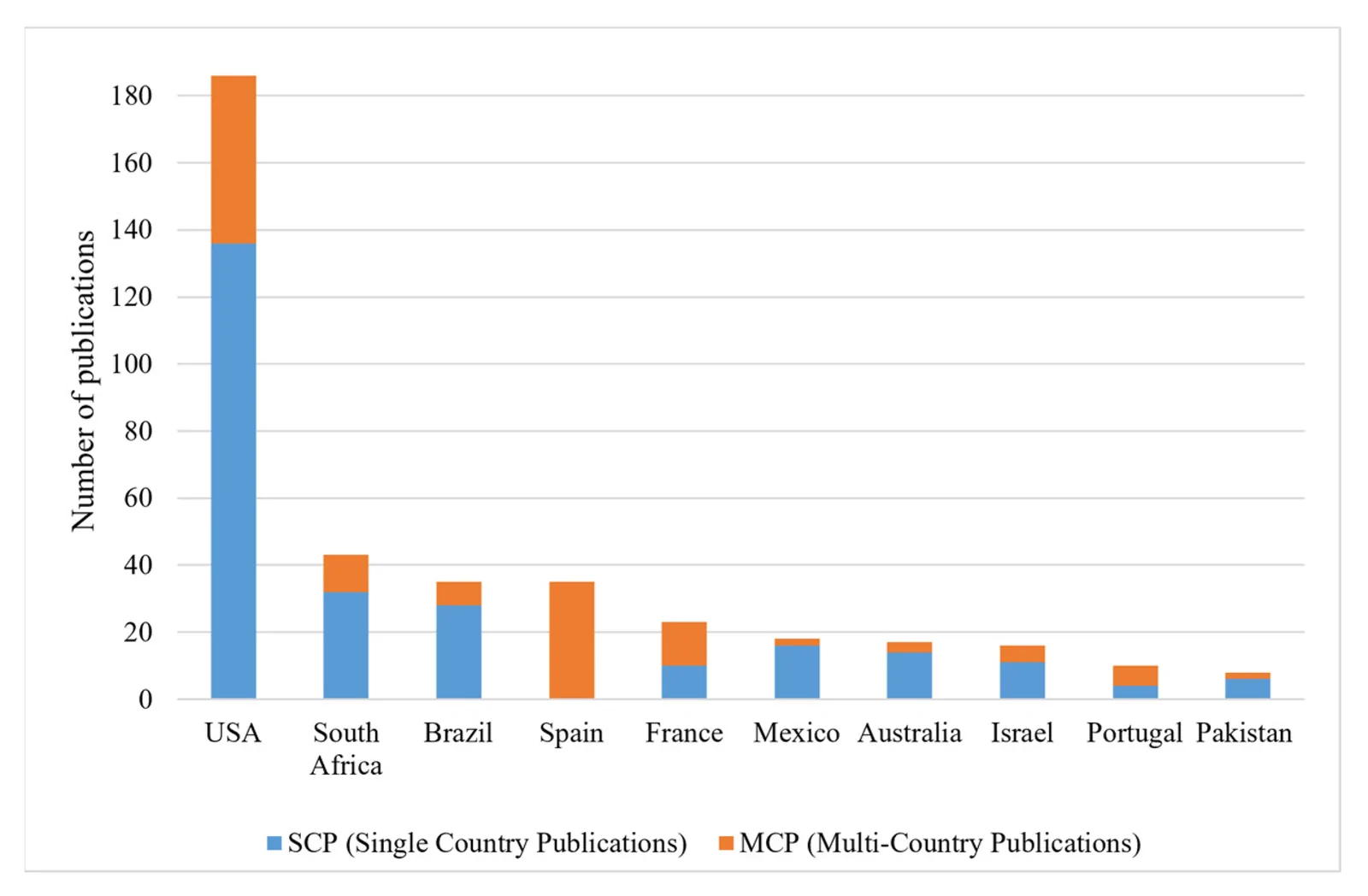
Top 10 countries contributing to vaccine research, shown by single-country publications (SCP) and multi-country publications (MCP).

**Figure 5:**
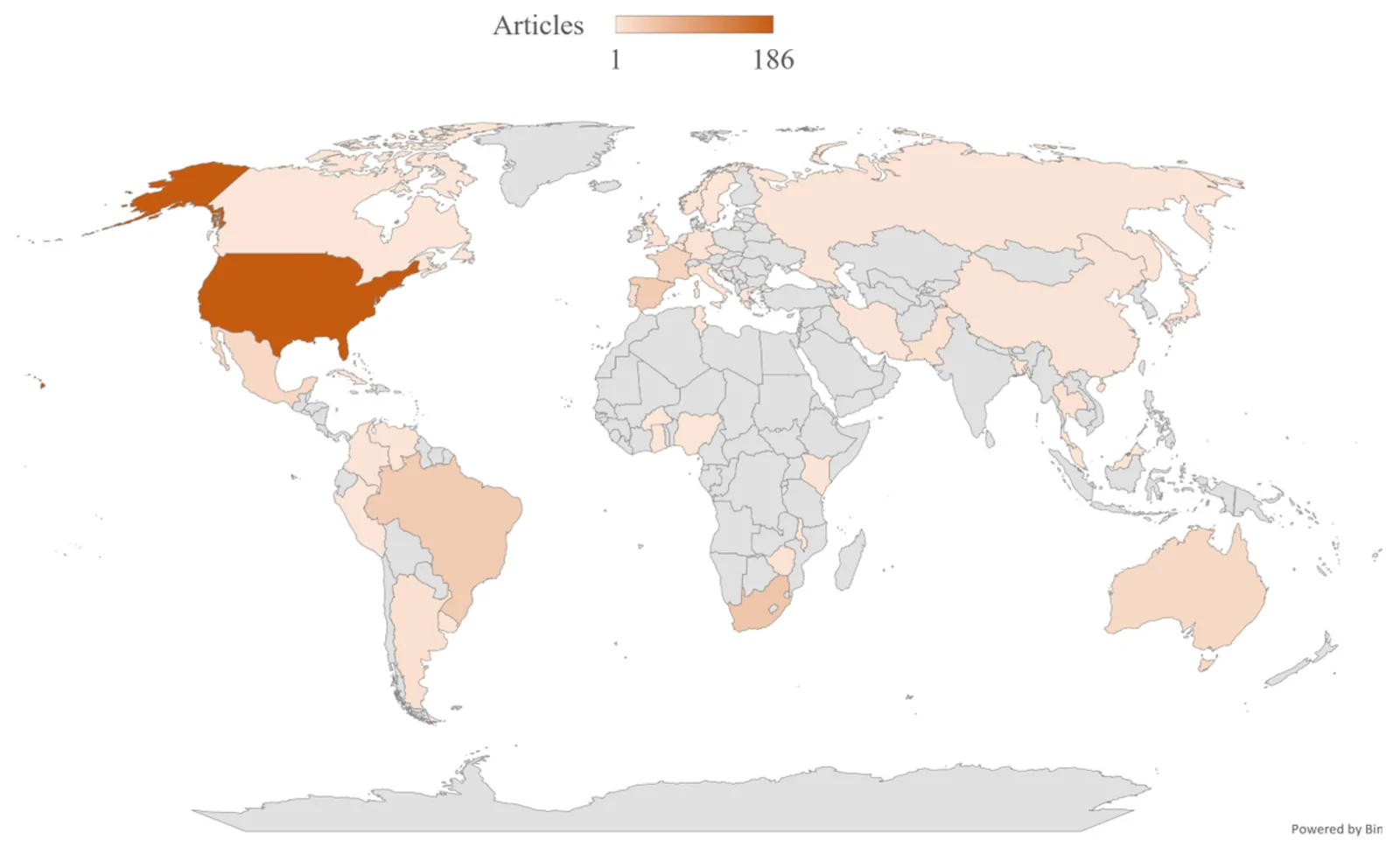
Global distribution of the countries contributing to vaccine research.

Publications on *Ehrlichia* and *Anaplasma* vaccine research originated from 44 countries, indicating broad international participation in this field (Figure 4 and Figure 5). However, most publications were contributed by a relatively small number of countries. The United States contributed the highest number of publications (*n* = 186, 38.5%), followed by South Africa (*n* = 43, 8.9%), Brazil and Spain (*n* = 35, 7.2%), and France (*n* = 23,4.76%). Additional contributions were observed from Mexico (*n* = 18, 3.7%), Australia (*n* = 17, 3.5%), Israel (*n* = 16, 3.3%), Portugal (*n* = 10, 2.1%), and Pakistan (*n* = 8, 1.7%).

The collaboration profile showed clear differences in the balance between single-country publications (SCP) and multi-country publications (MCP) among the leading countries (Figure 4). The United States had the highest output, with 136 SCPs (73.1%) and 50 MCPs (26.9%). South Africa had 32 SCPs (74.4%) and 11 MCPs (25.6%), while Brazil had 28 SCPs (80%) and 7 MCPs (20%). In contrast, all publications from Spain were multi-country publications (*n* = 35, 100%). France (*n* = 13, 56.5%) and Portugal (*n* = 6, 60%) also showed relatively high proportions of MCPs, indicating a stronger reliance on international collaboration rather than on single-country output. The global distribution map further showed that published studies were distributed across North America, South America, Europe, Africa, Asia, and Australia, indicating worldwide research interest in these pathogens (Figure 5).

The observed geographic distribution reflects the known veterinary and public health importance of *Ehrlichia* and *Anaplasma* infections in different regions of the world [6,37,38]. Countries with higher publication output may therefore represent areas where these pathogens are of greater clinical or economic relevance in the health of animals and humans. Overall, these findings suggest that research on *Ehrlichia* and *Anaplasma* vaccines has global participation, although scientific output remains led by a relatively small number of countries, with both national productivity and international collaboration shaping the field.

### 3.4. Major Institutions, Authors and Publication Sources

**Figure 6:**
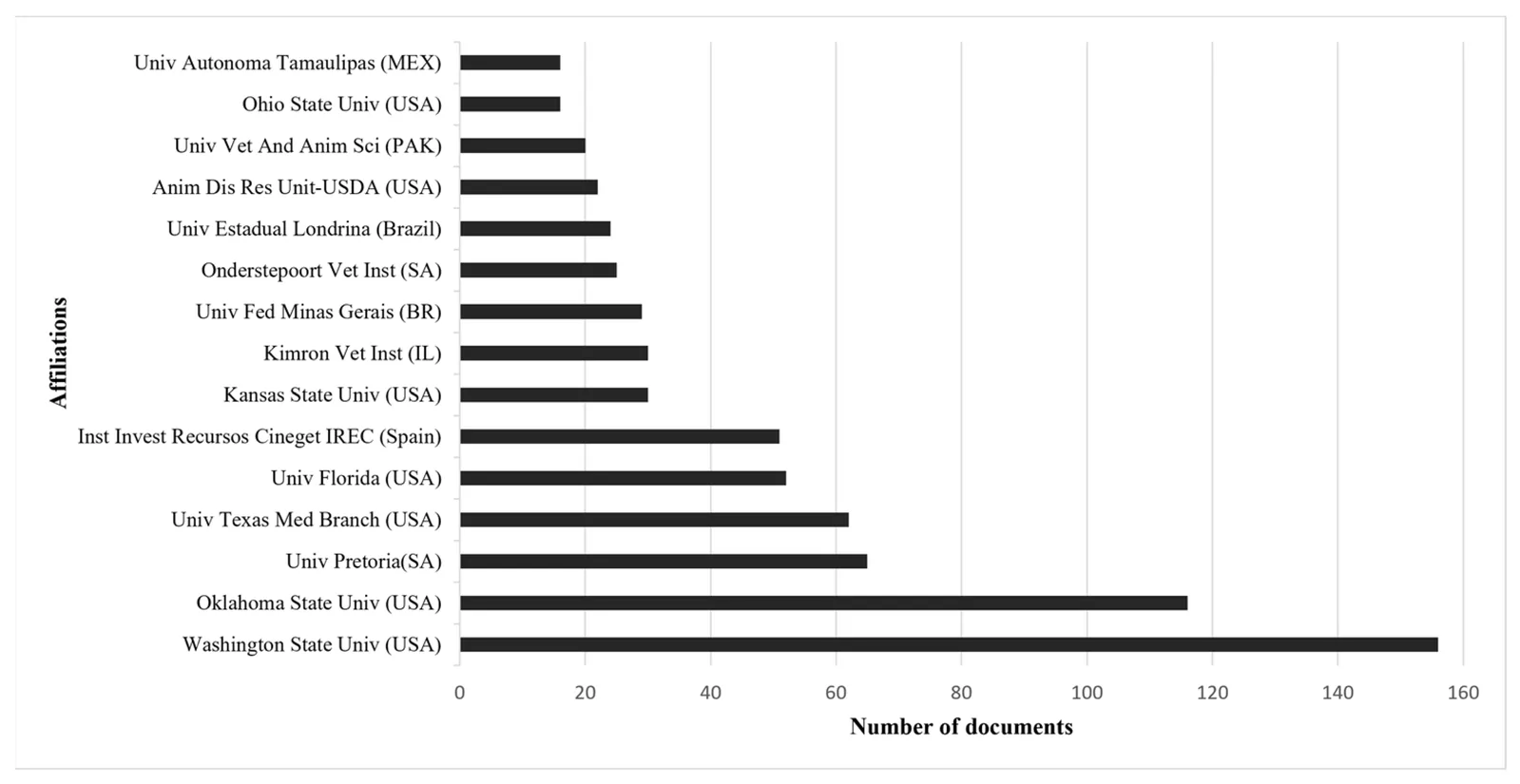
Institutions contributing to *Ehrlichia* and *Anaplasma* vaccine research ranked by the number of publications.

Institutional contributions to Ehrlichia and Anaplasma vaccine research were distributed across universities, veterinary institutes, and research centers in several countries (Figure 6). Washington State University (USA) and Oklahoma State University (USA) had the highest number of publications *n* = 156 (32.3%) and *n* = 116 (24.0%), respectively, followed by the University of Pretoria (South Africa) (*n* = 65, 13.5%), the University of Texas Medical Branch (USA) (*n* = 62, 12.8%), and the University of Florida (*n* = 52, 10.8%). This institutional distribution aligns with the geographic distribution observed in Figure 5, where the United States had the highest publication activity followed by South Africa and Brazil.

The author dataset included a total of 1809 authors, indicating that the literature reflects contributions from many researchers, although some authors appeared more frequently within the field. This author pattern aligns with the institutional distribution, as several frequently appearing authors have been affiliated with institutions that also showed high publication output, including Washington State University and Oklahoma State University, and these institutions have research programs dedicated to tick-borne diseases.

**Figure 7:**
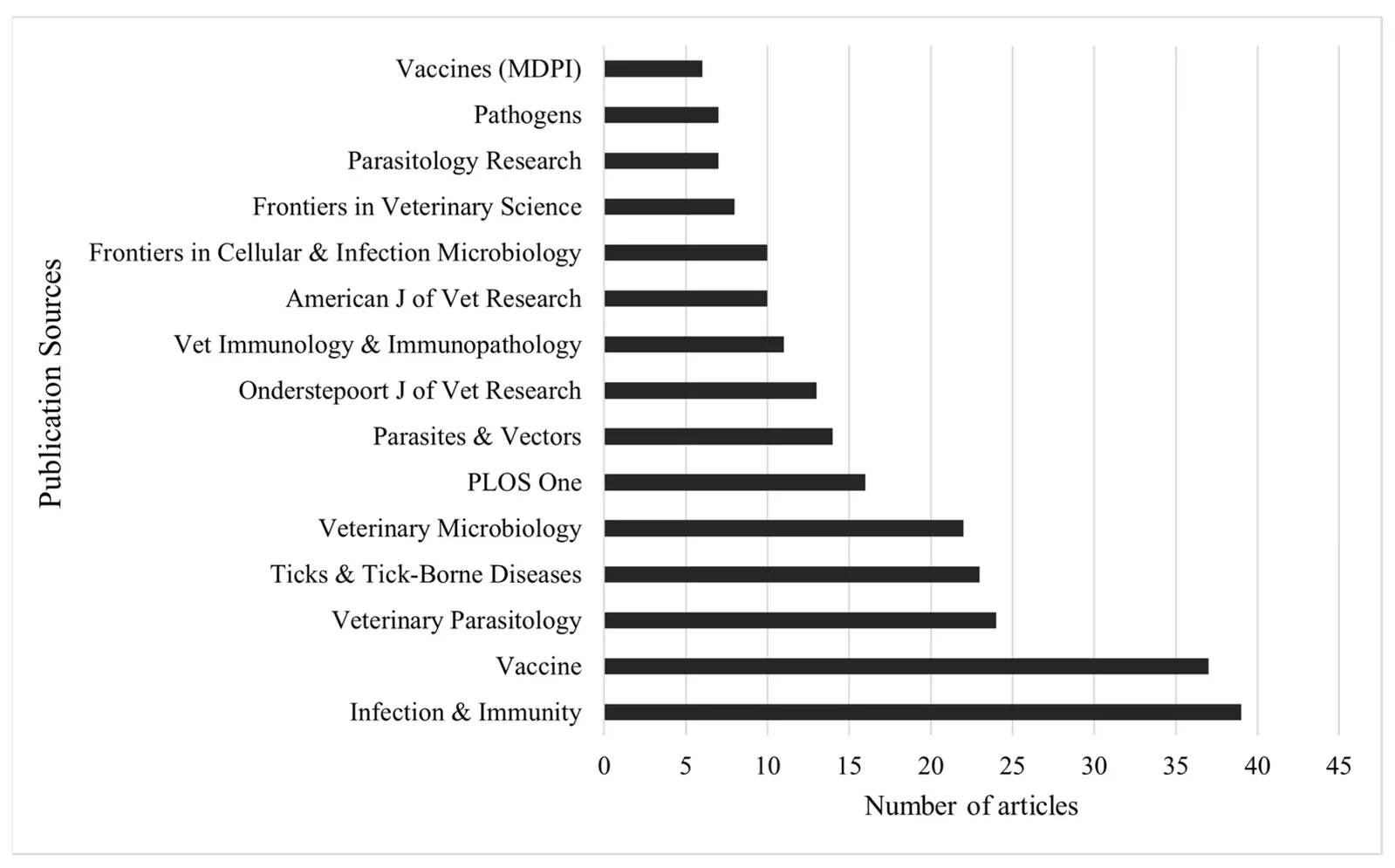
Most used publication sources for *Ehrlichia* and *Anaplasma* research ranked by number of articles.

The publications were distributed across 161 sources, indicating that this research has appeared in a wide range of journals (Figure 7). Infection and Immunity published the highest number of articles (*n* = 39, 8.1%), followed by *Vaccine* (*n* = 37, 7.7%), Veterinary Parasitology (*n* = 24, 5.0%), Ticks and Tick-borne Diseases (*n* = 23, 4.8%), and Veterinary Microbiology (*n* = 22, 4.6%). Overall, the main publication sources indicate that this field has been represented primarily in journals focused on microbiology, immunology, parasitology, and veterinary science. This distribution is consistent with the scope of the field, as vaccine-related studies are expected to appear in journals such as Vaccine, while Infection and Immunity reflect the importance of host immune responses in vaccine research. Similarly, journals such as Ticks and Tick-borne Diseases, Veterinary Parasitology, and Veterinary Microbiology reflect the tick-borne and veterinary relevance of these pathogens.

### 3.5. Keyword Frequency

**Figure 8:**
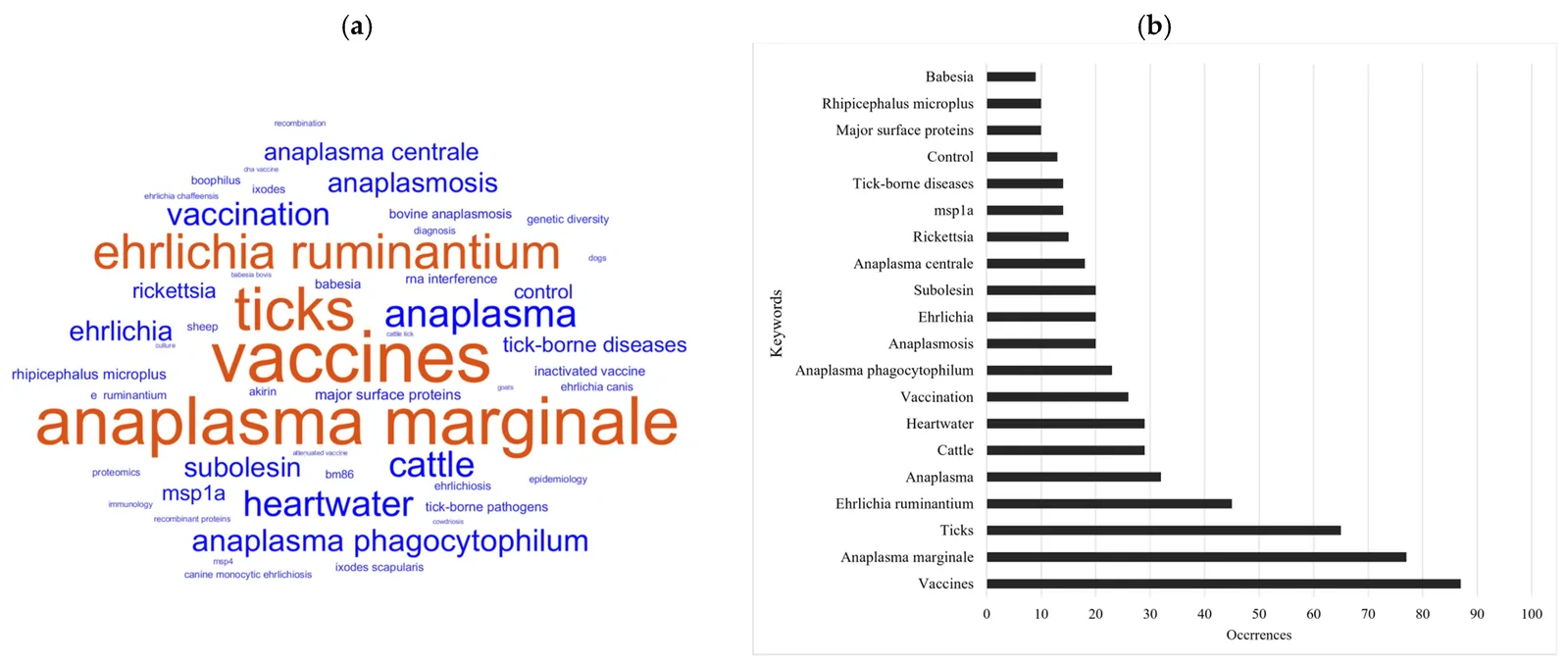
Keyword frequency analysis in *Ehrlichia* and *Anaplasma* vaccine research: (**a**) word cloud of the 50 most frequent words and (**b**) top 20 keywords by occurrence.

Keyword frequency analysis using author-provided keywords provided an overview of the main topics represented in *Ehrlichia* and *Anaplasma* vaccine research (Figure 8a,b). A total of 849 author keywords were included in the analysis. Beyond the expected vaccine-related terms, the literature focused mainly on a limited number of recurring pathogens and disease-related terms. *Anaplasma marginale* (*n* = 77) was the most frequent species-level keyword, followed by *Ehrlichia ruminantium* (*n* = 45) and *Anaplasma phagocytophilum* (*n* = 23). The frequent appearance of terms such as cattle (*n* = 29), heartwater (*n* = 29), and anaplasmosis (*n* = 20) indicates that a substantial part of the literature has focused on economically important livestock-associated infections. Keywords including subolesin (*n* = 20), msp1a (*n* = 14), major surface proteins (*n* = 10), Bm86 (*n* = 8), and RNA interference (*n* = 9) further suggest that research has also been focused on antigen discovery and vaccine design. Together, these terms indicate that the field has been shaped not only by species-specific vaccine studies, but also by antigen discovery and vector-associated control approaches.

### 3.6. Keyword Co-Occurrence Analysis

**Figure 9:**
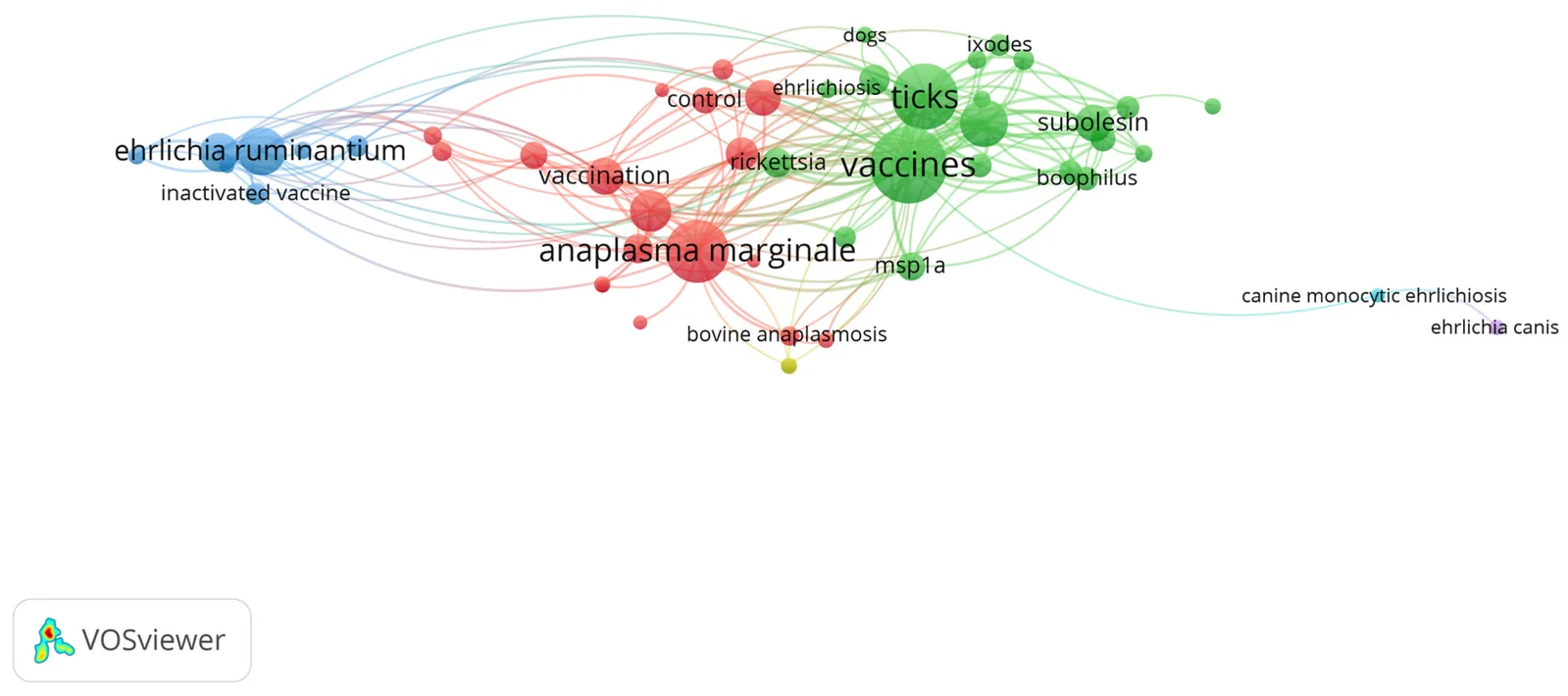
Keyword co-occurrence network showing the main thematic clusters in *Ehrlichia* and *Anaplasma* vaccine research. Larger nodes indicate keywords with higher frequency; smaller nodes indicate less frequent words, and lines between nodes represent co-occurrence relationships between terms within the same publications. Each color represents groups of keywords that are more closely related within the network.

Keyword co-occurrence analysis was used to examine how the main keywords were connected within the literature and to identify major research themes in the field. The largest and most central nodes were vaccines, ticks, and *Anaplasma marginale*, indicating that these topics formed the core of the literature. The network also showed strong links between pathogen names, disease terms, and antigen-related keywords (Figure 9).

Three major thematic clusters were identified. The first cluster was centered on *Anaplasma marginale*, vaccination, control, and bovine anaplasmosis, indicating a strong focus on bovine anaplasmosis and vaccine development in that area. A second cluster grouped vaccines, ticks, subolesin, msp1a, boophilus, and other tick-related terms, suggesting that part of the literature has focused on tick-associated antigens and vector-related control strategies. A third cluster was associated with *Ehrlichia ruminantium*, inactivated vaccine, and heartwater-associated terms, indicating work centered on heartwater vaccine development.

Smaller peripheral nodes, including ehrlichiosis, canine monocytic ehrlichiosis, and *Ehrlichia canis,* were less connected to the main clusters, suggesting that these topics were represented in the literature but were less central than the dominant themes related to bovine anaplasmosis, heartwater, and vector-associated vaccine strategies.

Antigen-related terms also frequently appeared in the vaccine literature, with keywords such as msp1a, major surface proteins, subolesin, and Bm86. However, only a subset of these antigens appeared in the keyword network.

## 4. Discussion

This bibliometric review maps the progression and research landscape of vaccine-related research on *Ehrlichia* and *Anaplasma*, highlighting key research trends and patterns over the past several decades. Overall, the publication pattern suggests gradual growth rather than rapid expansion. The increase in publications from the late 1990s onward may reflect improved molecular detection tools, increased recognition of tick-borne diseases in both veterinary and human medicine [39,40,41,42,43], and the expansion of genomic and proteomic approaches that facilitated antigen discovery and vaccine research [21,27]. While increased scientific interest and expanded funding for tick-borne diseases likely played a role, these factors remain speculative as funding data were not explicitly evaluated in this study.

A major finding was the stronger representation of bovine-associated pathogens and livestock-focused research themes compared with human and companion-animal infections. The prominence of *Anaplasma marginale*, bovine anaplasmosis, cattle, *Ehrlichia ruminantium*, and heartwater-related terms suggests that vaccine research has focused mainly on pathogens of major veterinary and economic importance. This likely reflects the impact of these diseases on animal health and livestock production, the strong veterinary and agricultural research base surrounding livestock diseases, and the long-standing need for practical control strategies in cattle and ruminants [12,13]. In contrast, pathogens such as *Ehrlichia chaffeensis*, *Anaplasma phagocytophilum*, and *Ehrlichia canis* were less prominent. However, keyword prominence does not necessarily capture the full representation of pathogens across the dataset.

Scientific output was also concentrated within a relatively small number of countries and institutions. The largest contributions came from the United States, South Africa, Brazil, Spain, and France, with major institutional hubs including Washington State University, Oklahoma State University, the University of Pretoria, the University of Texas Medical Branch, and the University of Florida. This pattern likely reflects both endemic disease relevance and research capacity. For example, South Africa is a major center for heartwater research because *Ehrlichia ruminantium* is endemic in much of sub-Saharan Africa and remains important in ruminants [13]. Likewise, Brazil and other countries in the Americas are closely linked to bovine anaplasmosis research because *A. marginale* is widely distributed and economically important in cattle-producing regions [12,15]. The strong contribution of the United States may reflect established veterinary and biomedical research programs, and long-standing interest in tick-borne and foreign animal diseases, including non-endemic pathogens such as *Ehrlichia ruminantium* [44].

The collaboration and journal patterns further show that this field has developed through both national research programs and international partnerships. The United States showed substantial single-country output, whereas countries such as Spain, France, and Portugal relied more heavily on multi-country collaborations. Leading publication sources also spanned microbiology, immunology, parasitology, veterinary medicine, and tick-borne disease research, highlighting the multidisciplinary nature of vaccine development for *Ehrlichia* and *Anaplasma.* Overall, the field appears to be shaped by a limited number of established research centers, local disease priorities, and broader scientific infrastructure.

The keyword network showed recurring antigen-related terms, suggesting that vaccine research has focused mainly on a limited number of commonly studied targets, particularly surface proteins, outer membrane-associated antigens, and host-interaction proteins. However, only some antigen targets appeared prominently in the network. This may be because bibliometric analyses capture terms that are repeatedly used as author keywords, while other vaccine candidates may be discussed within individual studies without appearing as author keywords [33,34]. The co-occurrence pattern also suggests that antigen-focused vaccine research has been more strongly concentrated in bovine anaplasmosis, heartwater, and tick-associated systems than across the full diversity of *Ehrlichia* and *Anaplasma* pathogens.

This pattern is consistent with published work highlighting *A. marginale* as a major focus of vaccine research [24] and with MSP1a extensively studied as a pathogen-derived surface antigen because of its immunogenicity, strain diversity, adhesion-related functions, and use in DNA vaccine and epitope-based vaccine studies [45,46,47,48]. In parallel, tick-associated antigens such as subolesin [49,50,51,52] and Bm86 [53] have been widely evaluated for vector-directed vaccine and tick control strategies. The *E. ruminantium* and heartwater-associated pattern is also consistent with the previous literature showing that heartwater control has long relied on vaccination and tick-control strategies. Vaccine research on *E. ruminantium* has focused on inactivated, attenuated, and antigen-based approaches; however, variable protection across strains remains an important challenge [13].

Across *Ehrlichia* and *Anaplasma* vaccine studies, a broad range of antigen targets have been reported; however, only a subset of these antigens appeared in the keyword network. In *Anaplasma marginale*, vaccine-related studies have examined outer membrane immunogens and major surface proteins, including MSP1a and other MSPs [54,55], as well as conserved outer membrane-associated candidates such as Am 779, Am854/OmpA, Omp7, Omp8, and Omp9 [55,56,57]. Recombinant candidates, including VirB9, VirB10, VirB11, and elongation factor-Tu, have also been evaluated, although one recombinant T4SS/Ef-Tu vaccine formulation did not protect cattle against virulent challenge [58]. In *Anaplasma phagocytophilum*, recombinant MSP4 and MSP4-HSP70 antigens, as well as recombinant Asp14 and OmpA, have been investigated as vaccine candidates in sheep [59,60]. Adhesin-binding domains from Asp14, OmpA, and AipA have also been evaluated in a mouse vaccination model and were shown to confer protection against infection [61].

In *Ehrlichia ruminantium*, MAP1 has been evaluated as a vaccine candidate using DNA vaccination and recombinant protein-boost strategies [62,63], although antigenic diversity among MAP1 family proteins remains an important consideration for heartwater vaccine development [64]. Multigene DNA vaccine approaches have also tested open reading frame cocktails, such as 1H12 in sheep [65,66]. In *Ehrlichia canis*, recombinant GP19 has been evaluated as a vaccine prototype in a mouse model [67]. In *Ehrlichia chaffeensis*, EtpE has been evaluated as an entry-triggering protein vaccine candidate against tick-transmitted infection [68], while OMP-1B and type IV secretion-associated VirB2-4 have been tested in dogs for protection against tick-transmitted *E.chaffeensis* challenge [69].

Although antigen-based approaches were prominent, they are not the only vaccine strategies explored in this field. Attenuated and genetically modified live vaccine approaches have been evaluated for *Ehrlichia canis* [29,70], attenuated approaches have been evaluated for *Ehrlichia ruminantium* [71,72,73], and genetically modified live vaccine approaches have been tested for *Ehrlichia chaffeensis* [74,75,76,77] and *Anaplasma marginale* [78,79]. These approaches did not emerge as dominant keyword clusters in the present analysis, which may reflect differences in keyword use rather than their absence from the vaccine literature. The relatively low centrality of *E. canis* is also consistent with the limited vaccine literature for canine monocytic ehrlichiosis [27]. However, recent studies identifying antibody-reactive proteins, immunoreactive antigens, and selected vaccine candidates suggest that *E. canis* antigen discovery may support future vaccine development [80,81].

Despite advances in identifying candidate antigens and evaluating attenuated and genetically modified vaccine approaches, several challenges remain across these pathogens. Antigenic and strain diversity can limit cross-protective immunity, while the obligate intracellular nature and complex interactions with vertebrate and tick hosts complicate the identification of protective immune responses and broadly effective vaccine targets. The extent and diversity of vaccine research have varied among species, with some pathogens having multiple antigen and live vaccine approaches under investigation, while research on other species remains comparatively limited.

Together, these findings suggest several directions for future research. First, the comparatively lower representation of several *Ehrlichia* and *Anaplasma* pathogens, particularly those relevant to human and companion-animal health, identifies areas where further assessment is needed to determine whether lower research activity reflects gaps in vaccine research, differences in disease burden, funding patterns, or other research priorities. Second, stronger integration of vector biology, host immune responses, and pathogen genomics may help address persistent challenges in antigen discovery and vaccine design. Third, increased international collaboration, especially in endemic but underrepresented regions, may help address geographic and pathogen-related gaps. Finally, more consistent use of informative keywords may improve the visibility of emerging research areas within the field. Thus, the practical value of this bibliometric analysis is to identify where research activity has historically been concentrated or comparatively limited and to provide a quantitative framework for future assessments that integrate publication patterns with epidemiological, disease-burden, economic, and funding data.

This study should be interpreted in the context of several limitations. The analysis was restricted to the Web of Science database, so relevant publications indexed in other databases may not have been captured [35]. The results also depend on the selected search terms and the accuracy of bibliographic metadata, including author-provided keywords, which may not fully represent all concepts or pathogens addressed in individual studies. In addition, bibliometric indicators reflect publication and citation patterns rather than direct measures of scientific quality, vaccine efficacy, or experimental success [34,82]. Citation patterns should also be interpreted cautiously because citation counts may be influenced by factors such as publication age, the number of publications produced over time, and the number of references typically cited in scientific articles over time. Although the average citations per year metric accounts for differences in the time available for publications to accumulate citations, it does not account for all factors that may influence citation patterns across the study period. Accordingly, the current study was not designed to systematically compare vaccine efficacy, protective outcomes, or stages of vaccine development across individual species. Similarly, lower representation in the published literature should not be interpreted as evidence of underinvestment or lower research priority, because the current analysis did not evaluate disease burden, funding levels, or public health and veterinary impact. Despite these limitations, this analysis provides a structured overview of how vaccine research on *Ehrlichia* and *Anaplasma* has evolved and identifies key areas where research efforts have been concentrated, as well as areas that remain comparatively less represented in the published literature and may warrant future investigation.

## Figures and Tables

**Table 1:** Data description showing number of sources, number of documents along with other characteristics.

Description	Value
Timespan	1965:2025
Sources (journals, books, etc.)	161
Total number of documents	483
Annual growth rate, %	4.08
Document average age (mean ± SD (years))	15.7 ± 11.1 years
Average citations per document (mean ± SD)	27.1 ± 39.02
Keywords plus (ID) *	1290
Author keywords (DE) *	849
Total number of authors	1809
Single-authored documents	25 (5.2%)
Co-authors per document (mean ± SD)	6.46 ± 3.80
International co-authorships, %	173 (35.82%)

* ID = Keywords plus, database-generated keywords from Web of Science; DE = author keywords, keywords provided by the authors of the article.

## Data Availability

The original contributions presented in this study are included in the article/Supplementary Material. Further inquiries can be directed to the corresponding author.

## Supplementary Materials

The following supporting information can be downloaded at https://www.mdpi.com/article/10.3390/microorganisms14091967/s1. Table S1: Reported species of *Ehrlichia* and *Anaplasma*, associated diseases, host cell tropism, and host range. References [5,8,83,84,85,86,87,88,89,90,91,92,93,94,95,96,97,98,99,100,101,102,103,104,105,106,107,108,109,110,111,112,113,114,115,116,117,118] are cited in the Supplementary Materials.
