# Supplementary material for: Global Trends in Vaccine Research on Ehrlichia and Anaplasma: A Bibliometric Review
Source: Microorganisms. 2026 Sep 6;14(9):1967. doi: 10.3390/microorganisms14091967 (PMC13608919; doi:10.3390/microorganisms14091967)
Supplement: Supplementary file 1 [file microorganisms-14-01967-s001.zip › microorganisms-4552914-supplementary.pdf]

Table S1. Reported species of *Ehrlichia* and *Anaplasma*, associated diseases, host cell tropism, and host range

Species	Disease	Cell Tropism	Host species affected	Ref
Ehrlichia chaffeensis	Human monocytic ehrlichiosis	Monocytes/macrophages	Humans, dogs	(83-85)
E. ewingii	Granulocytic ehrlichiosis	Neutrophils/granulocytes	Dogs, humans	(86, 87)
E. canis	Canine monocytic ehrlichiosis	Monocytes/macrophages	Dogs; occasional human infections reported	(88-92)
E. ruminantium	Heartwater	Endothelial cells	Cattle, sheep, goats, water buffalo	(8)
E. muris	Murine-ehrlichiosis like infection	Monocytes/macrophages	Wild mice; experimental infection of laboratory mice, Dogs	(93-95)
E.muris subsp *eauclairensis*	Human ehrlichiosis	Monocytes/macrophages	Humans	(96, 97)
E. minasensis	Emerging ehrlichial infection in cattle	Not clearly established *in vivo*; propagated in DH82 (macrophage-like) and tick cell lines	Cattle; also detected or reported in deer/cervids, goats, horses and dogs.	(98-101)
Panola Mountain Ehrlichia	Emerging tick-borne ehrlichiosis- like illness	Not clearly established	Goats, white-tailed deer, humans, dogs, and cattle	(102-104)
Anaplasma marginale	Bovine anaplasmosis	Erythrocytes	Cattle, goats, sheep, bison, and water buffalo	(105)
A. centrale	Mild bovine anaplasmosis; Historically used as a live vaccine strain	Erythrocytes	Cattle	(106)
A. ovis	Ovine Anaplasmosis	Erythrocytes	Sheep and goats	(107-109)

A. bovis	Monocytic anaplasmosis-like infection	Monocytes/macrophages	Cattle, goats, and sheep	(107)
A. phagocytophilum	Human granulocytic anaplasmosis, tick-borne fever in ruminants, and granulocytic anaplasmosis in dogs and horses	Neutrophils/granulocytes	Humans, dogs, and horses. sheep, and goats	(5, 110)
A. platys	Infectious canine cyclic thrombocytopenia	Platelets	Dogs	(111, 112)
A. capra	Emerging zoonotic anaplasmosis	Infection observed in erythrocytes; not clearly established	Goats, humans, sheep, and cattle	(113-118)
